# Accuracy of Weight Estimation Using the Broselow Tape in a Peruvian Pediatric Population

**DOI:** 10.7759/cureus.15807

**Published:** 2021-06-21

**Authors:** Jerry Z Oommen, Mark Hodgins, Rene Hinojosa, Gary Willyerd, Travis Gordon, John Ashurst, Joe Gorz, Santiago Benites, Ruben K Briceno, Shane Sergent

**Affiliations:** 1 College of Osteopathic Medicine, Michigan State University, East Lansing, USA; 2 Information Technology, Michigan State University, East Lansing, USA; 3 Institute for Global Health, Michigan State University, East Lansing, USA; 4 Emergency Medicine, Kingman Regional Medical Center, Kingman, USA; 5 Family Medicine, Michigan State University, East Lansing, USA; 6 Research, Universidad César Vallejo, Trujillo, PER

**Keywords:** broselow tape, weight estimation, peru, pediatrics, resuscitation

## Abstract

Introduction

The Broselow tape (BT) is a useful pediatric tool for weight estimation and dosing reference during emergency care. Many accuracy studies have been performed for various countries and regions of the world but there is very little information for Latin American countries. The primary objective of the study was to assess the accuracy of the BT in a Peruvian pediatric population.

Methods

This was a retrospective cross-sectional study of 1,160 children aged two to 19 years from three outpatient clinics in La Libertad, Lima, and Iquitos, Peru. Patient height and weight were measured and compared with the weight and color zone generated by the 2017 edition of the BT. Accuracy was estimated by statistical comparison of mean absolute percent differences, error within 10% (EW10), and color zone agreement.

Results

Comparison of mean differences between measured weight (MW) and estimated BT weight shows that the BT underestimates actual weight for all color zones in this population. Likewise, the Bland-Altman plot of agreement between estimated and measured weights shows an overall underestimation, or bias, equal to 1.60 kg. The overall percent difference was -7.84% with differences gradually increasing for weights over 10 kg. In terms of accuracy, the overall error within 10% was 62.8%.

Conclusion

The BT underestimates the actual weight of Peruvian pediatric patients in all color categories, particularly in children with higher body mass indexes. Underestimation of weight may lead to the use of non-therapeutic medication doses or incorrect equipment sizes and, subsequently, ineffective resuscitation.

## Introduction

Accurate weight estimation is a critical first step in the process of calculating a safe and effective medication dose for children. During a pediatric emergency, weighing a child is often not feasible due to a lack of equipment and the condition of the patient. These factors, coupled with a complex calculation [1], could explain why pediatric medication dosing errors occur in emergency settings [1]. In order to alleviate some of this burden, numerous weight estimation methods have been developed. One of the most widely used methods is the Broselow tape, which was developed in the United States based on data from the National Health and Nutrition Examination Survey [2]. The BT requires a provider to position the tape beside the patient and measure them from head to heel. This length translates to a weight estimation and color-coded zone that lists appropriate weight-based drug dosages, volumes, and equipment sizes.

As emergency medicine has grown globally, the utility of the BT has been tested in international populations with the hope of improving pediatric resuscitation. Assessment of the BT’s accuracy internationally has yielded mixed results. A recent systematic review reported that the BT tends to underestimate weight in high-income countries while overestimating in low- and middle-income countries [3]. This difference may be due to the variation in obesity rates, given that the BT was derived from United States pediatric data [2,4]. Despite widespread assessment of the BT, there has been only one study on the BT in Latin American and none in Peru [5]. Considering the prevalence of low- or middle-income countries and increasing obesity in Latin America, the utility of the BT remains unclear in this region [6]. This study aims to assess the accuracy of the BT in a Peruvian pediatric population.

A poster of this article was presented at the Bureau of International Osteopathic Medicine 2020 Abstract Competition on October 17, 2020, in Austin, TX.

## Materials and methods

The data for this retrospective cross-sectional study were collected from August 2014 to August 2018 from patients who visited outpatient clinics in La Libertad, Lima, and Iquitos, Peru. Participation was completely voluntary and parental consent was obtained for all participants. Children were excluded if they were > 19 years old, their height was < 46.6 or ≥ 143.3 cm (BT limits), or if they were not Peruvian. This study was approved by the Michigan State University and Universidad Cesar Vallejo Institutional Review Boards.

Data collected included height, weight, age, sex, and date of birth. Participant height was measured using a field anthropometer (model No. 601, Seritex Inc., Rutherford, NJ). Two independent measurements were performed with an accepted error of 0.3 cm. Measured weight (MW) was measured to the nearest 0.1 kg using a body composition monitor (BC-534 InnerScan Body Composition Monitor, Tanita Corporation of America, Inc., Arlington Heights, IL) with clothing and shoes removed.

Measured height was converted to tape weight (TW) and color zone according to the BT (Broselow Pediatric Emergency Tape 2017 Edition A, Armstrong Medical Industries, Lincolnshire, IL). Body mass index (BMI) was calculated using the standard formula ($BMI = kg/m2$), and overweight categorization of children was assessed using the Center for Disease Control’s (CDC) Year 2000 BMI-for-age chart [7].

Pearson’s correlation and linear regression were used to assess the relationship between MW and TW, along with t-tests to determine the significance of the difference between the two measurements. Accuracy was assessed by Bland-Altman plots, zone agreement, mean absolute percent error, and error within 10%. Bland-Altman plots were used to compare TW to MW in order to obtain bias and the limits of agreement between the measurements. Zone agreement was defined as the percentage of times the predicted color zone (determined based on patient height) matched the true color zone (determined based on MW). The percentage of MW that fell one color zone above the estimated color zone was also calculated. Mean absolute percent error (MAPE) was the degree of deviation of the measured weight from the actual weight reported as a percentage. EW10 was defined as the percentage of TW that fell 10% above or below the MW. A 10% cut-off was used, as it is a widely accepted degree of error. Subgroup analysis was also done in order to make comparisons by age and gender.

In order to improve BT accuracy, curve fitting techniques were applied to the observed data. The different options tested were growth and regression equations and readjustment of color based on successive approximations. All statistical analysis was performed using SPSS version 26 (IBM Corp, Armonk, NY).

## Results

A total of 1,160 children are included in this study, of which 51% were male and 49% were female. The mean age, MW, and TW of participants were 5.8 ± 3.7 years, 20.52 ± 9.7 kg, and 18.91 ± 8.08 kg, respectively (Table 1).

**Table 1:** Demographic characteristics of study participants N: sample size; kg: kilogram; cm: centimeter; BMI: body mass index

Demographic Traits	Male	Female	Total
N	593 (51%)	567 (49%)	1160
Age (years)	5.56	6.05	5.80 ± 3.69
Measured Weight (kg)	20.08	20.98	20.52 ± 9.70
Height (cm)	104.03	105.71	104.85 ± 23.06
BMI	17.50	17.60	17.54 ± 2.69
6 to 11 years old	17.81 ± 2.72 (36.5%)	17.75 ± 2.52 (35.6%)	--
12 to 18 years old	19.84 ± 3.03 (25.0%)	20.96 ± 3.15 (20.5%)	--
Tape Weight (kg)	18.57	19.27	18.91 ± 8.08

Assessment of BMI by age group and gender demonstrates an increase of BMI with age and is similar for both males and females. However, a larger proportion of overweight children are present in those aged six to 11 years (36%) compared to those 12-18 years (20%-25%) (Table 1).

**Figure 1:**
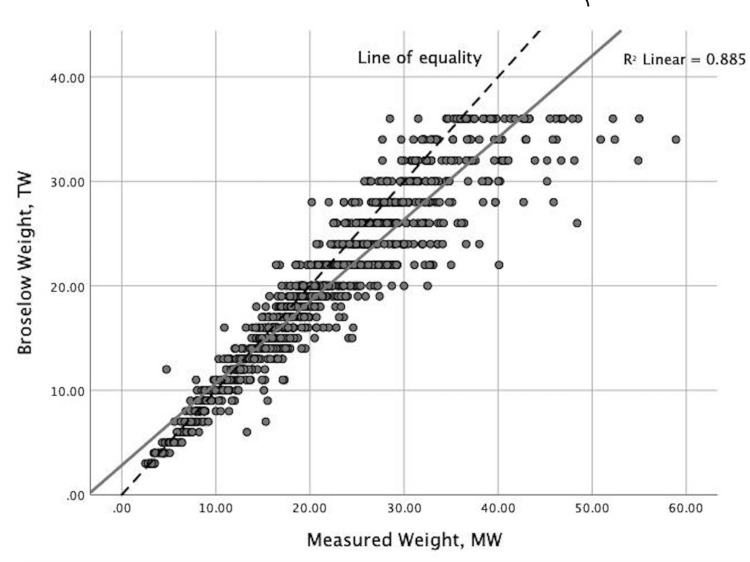
Correlation between tape weight and measured weight

The data were further analyzed to determine the relationship between measured and estimated weights. Figure 1 illustrates the strong positive correlation found between the two variables (r = 0.941, p < 0.01). The variation of the BT was explored by calculating the mean difference between TW and MW across each color zone (Table 2). The findings indicate that the mean difference is negative or underestimates weight in all color zones with the largest mean difference occurring in the heaviest weight zone (green, -3.74 kg), and the smallest mean difference occurring in the lightest weight zone (gray, -0.25 kg).

**Table 2:** Comparison of Broselow tape weights and measured weights by color zone N: sample size; TW: tape weight; MW: measured weight; SD: standard deviation

Color Zone	N	TW ± SD	MW ± SD	TW - MW	% Difference
Gray	47	4.128 ± 0.824	4.375 ± 1.039	-0.247	-5.65%
Pink	38	6.579 ± 0.500	7.555 ± 1.827	-0.976	-12.92%
Red	61	8.525 ± 0.503	8.80 ± 1.363	-0.275	-3.13%
Purple	84	10.464 ± 0.502	10.971 ± 1.877	-0.507	-4.62%
Yellow	130	13.139 ± 0.734	13.839 ± 1.918	-0.700	-5.06%
White	220	16.441 ± 1.155	17.396 ± 2.208	-0.955	-5.49%
Blue	257	20.720 ± 1.281	22.594 ± 3.551	-1.874	-8.29%
Orange	173	26.00 ± 1.628	28.395 ± 4.464	-2.395	-8.43%
Green	150	32.973 ± 2.360	36.709 ± 6.547	-3.736	-10.18%
Total	1160	18.911 ± 8.078	20.519 ± 9.695	-1.608	-7.84%

However, correlation and variation do not convey the accuracy of the BT. To assess accuracy multiple methods were used, including the Bland-Altman plot with 95% confidence interval, zone agreement, mean absolute percent error, and error within 10%.

**Figure 2:**
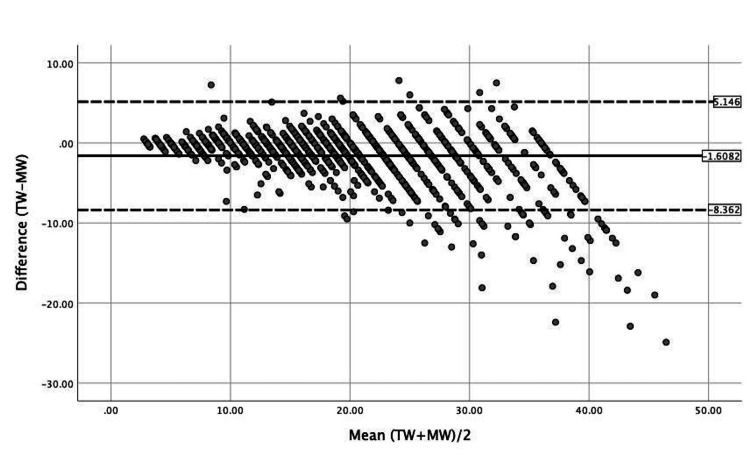
Bland-Altman plot of agreement

The Bland-Altman plot demonstrates a bias equal to -1.61 kg with limits of agreement of -8.36 and 5.15 (Figure 2). This figure demonstrates that many observation points fall outside the LOA in weights over 19 kg, indicating there is not a significant degree of agreement for children with heavier weights.

Table 3 demonstrates accuracy in terms of zone agreement. The greatest % agreement in the lightest color zone (gray, 85.1%) and lowest % agreement in the heaviest color zone (green, 42.6%). Looking at the % of MW that exceeded the estimated color zone, the smallest value was observed in the lightest color zone (12.8%), and the greatest value was observed in the heaviest color zone (47.3%), consistent with the previous findings suggesting that higher variations occurred in heavier weights.

**Table 3:** Broselow tape accuracy measured by percent zone agreement and percent next zone higher

Color Zone	% Zone Agreement	% Next Higher Zone
Gray	85.1	12.8
Pink	68.4	26.3
Red	75.4	13.1
Purple	52.4	20.2
Yellow	66.1	26.2
White	68.2	24.1
Blue	52.9	33.8
Orange	54.9	32.9
Green	42.6	47.3

Another measure of accuracy assessed is the MAPE shown in Table 4. The MAPE ranges from 8.27% (white, 15-18 kg) to 12.51% (pink, 6-7 kg), with an overall error of 10.11%. Despite the largest MAPE being present in the pink color zone, the general trend is that errors increase as weight increases. A second means of looking at MAPE is EW10 (Table 4). Overall, EW10 is 62.8%, with individual color zones ranging from 54.7% (green) to 75.4% (red). This further conveys that the BT is more accurate at lighter weights and less accurate at heavier weights.

**Table 4:** Accuracy measured by mean absolute percent error and error within 10% for the Broselow tape and alternative weight estimation models BT: Broselow tape; AT: adjusted tape; GC: growth curve; RA: regression analysis; MAPE: mean absolute percent error; EW10: error within 10 percent

Color Zone	BT MAPE	BT EW10	AT MAPE	AT EW10	GC MAPE	GC EW10	RA* MAPE	RA* EW10
Grey	9.08	59.6	8.00	63.4	33.36	14.9	32.76	2.1
Pink	12.51	55.3	10.62	67.5	10.10	71.1	25.26	2.6
Red	8.61	75.4	9.32	67.7	8.89	73.8	13.95	39.3
Purple	9.89	58.3	9.58	57.7	11.86	52.4	9.89	69.0
Yellow	9.36	70.0	10.56	68.6	12.05	56.9	10.33	63.6
White	8.27	69.5	8.44	73.3	9.5	64.1	11.19	54.1
Blue	10.49	61.1	8.31	71.4	9.63	66.5	12.11	51.0
Orange	11.13	59.0	10.75	55.9	10.06	61.8	10.96	61.3
Green	12.05	54.7	11.84	53.8	11.16	62.0	11.36	51.3
Total	10.11	62.8	9.81	64.0	11.24	61.1	12.66	51.7

Based on the observed findings, multiple models to improve weight estimation accuracy were explored, including a multi-variable linear regression, growth curve, and BT adjustment based on successive approximation. Comparisons between all the proposed methods were assessed and demonstrated in Table 4. The overall accuracy, in terms of EW10, decreases in both the regression model (51.7%) and growth curve (61.1%) but marginally increases with the AT (64%). For the AT, the MAPE improves for 6/9 color zones with an overall value of 9.81, compared to the overall MAPE of 10.11 seen in the BT. Based on the EW10 and MAPE, the adjusted tape was shown to be more accurate than the BT for this population.

## Discussion

Recently, researchers have worked to ascertain the BT’s utility in pediatric populations across the globe as length-based weight estimation tools are endorsed by major advanced life support groups [8]. This is the first study of the BT conducted in Peru, and it demonstrates that the BT underestimates weight by an average of 1.61 kg (7.84%) and the EW10 is 62.8%. This is in line with a study conducted in Mexico, which noted the BT underestimated weight, but in disagreement with the findings of a systematic review, regarding low- and middle-income countries [3,5]. These variations in BT estimation worldwide highlight the importance of evaluating the BT in the population of interest prior to implementation.

Given that the BT is intended to predict ideal body weight based on height, one factor contributing to the underperformance of the BT in this study may be the prevalence of obesity. The prevalence of overweight and obese children has increased worldwide, from 4% in 1975 to about 18% in 2016 [9]. Similar trends hold true for Latin America and in multiple regions of Peru [4,10]. In accordance with existing data, a significant proportion of the study population was found to be overweight and obese with 36% of children six to 12 years and 20%-25% 13-18 years meeting these criteria.

In order to account for such limitations of the BT, particularly in heavier children, previous studies recommended the use of tape modifications. These include quantitative correction factors or qualitative adjustments based on body habitus assessment [11-12]. Here the adjusted tape, created based on successive approximation by adjusting the height ranges using the observed data, demonstrates a slight overall improvement in EW10 from 62.8% to 64%. The specific changes to the tape, illustrated in Table 5, improves prediction in zones where the BT is less accurate and better reflects the pediatric population of Peru.

**Table 5:** Comparison of height and weight ranges between Broselow and adjusted tapes BT: Broselow tape; AT: adjusted tape; cm: centimeter; kg: kilogram

Color Zone	BT Height (cm)	BT Weight (kg)	AT Height (cm)	AT Weight (kg)
Grey	46.6 - 59	3 - 5	46.6 – 57	3 - 5
Pink	59.0 – 66.7	6 - 7	57.0 – 66.0	6 - 7
Red	66.7 – 74.0	8 - 9	66.0 – 74.0	8 - 9
Purple	74.0 – 83.6	10 - 11	74.0 – 82.0	10 – 11
Yellow	83.6 – 95.2	12 - 14	82.0 – 92.0	12 – 14
White	95.2 – 108.0	15 - 18	92.0 – 104.8	15 – 18
Blue	108.0 – 121.2	19 - 22	104.8 – 116.0	19 – 22
Orange	121.2 – 130.4	24 - 28	116.0 – 128.8	24 – 28
Green	130.4 – 143.3	30 - 36	128.8 – 143.3	30 – 39

Limitations

Though this study was conducted at multiple sites, the population of patients seen in the outpatient clinics may not be necessarily a representative sample of the entire pediatric population of Peru. Given the study’s location, the population may not adequately represent the critically ill patient requiring resuscitation. Although uniformity was emphasized in the clinics, different team members were taking measurements that may have led to variations of measurements. Lastly, only one weight estimation tool, the 2017 BT, was evaluated, so no comparison to other versions of the BT or estimation tools can be made for this population.

## Conclusions

The findings of this study indicate that the Broselow tape underestimates weight in the Peruvian pediatric population, with larger errors for heavier populations. BMI analysis shows that the children are characterized by an increased prevalence of overweight and obesity categorization. Subtle modifications to the tape, based on the gathered population data, allowed for the development of an adjusted tape with enhanced accuracy and reliability. Similar studies are necessary throughout Latin America prior to widespread adoption for pediatric resuscitations.
